# Distance from the magnification device contributes to differences in lower leg length measured in patients with TSF correction

**DOI:** 10.1007/s00402-021-03831-1

**Published:** 2021-03-06

**Authors:** Marc-Daniel Ahrend, Michael Rühle, Fabian Springer, Heiko Baumgartner

**Affiliations:** 1 Department of Traumatology and Reconstructive Surgery, BG Trauma Center Tübingen, Eberhard-Karls University of Tübingen, Schnarrenberg-Str. 95, 72076 Tübingen, Germany; 2 AO Research Institute Davos, Davos, Switzerland; 3 Department of Diagnostic and Interventional Radiology, University Hospital Tuebingen, Tübingen, Germany

**Keywords:** Long-leg radiographs, TSF, External fixator, Limb length, Lengthening

## Abstract

**Introduction:**

In absence of deformity or injury of the contralateral leg, the contralateral leg length is used to plan limb lengthening. Length variability on long-leg weight-bearing radiographs (LLR) can lead to inaccurate deformity correction. The aim of the study was to (1) examine the variability of the measured limb length on LLR and (2) to examine the influence of the position of the magnification device.

**Materials and methods:**

The limb lengths of 38 patients during deformity correction with a taylor-spatial-frame were measured retrospectively on 7.3 ± 2.6 (4–13) LLR per patient. The measured length of the untreated limb between LLR were used to determine length variability between LLR in each patient. To answer the secondary aim, we took LLR from a 90 cm validation distance. A magnification device was placed in different positions: at the middle of the 90 cm distance (*z*-position), 5 cm anterior and 5 cm posterior from the *z*-position, at the bottom and top of the validation distance as well as 5 cm medial and 15 cm lateral from the *z*-position.

**Results:**

The measured length variability ranged within a patient from 10 to 50 mm. 76% of patients had a measured limb length difference of ≥ 2 cm between taken LLR. Compared to length measurement of the 90 cm test object with the magnification device in the *z*-position (90.1 cm), positioning the device 5 cm anterior led to smaller (88.6 cm) and 5 cm posterior led to larger measurements (91.7 cm). The measured length with the magnification device at the bottom, top, medial or lateral (90.4; 89.9; 90.2; 89.8 cm) to the object differed not relevantly.

**Conclusions:**

High variability of limb length between different LLR within one patient was observed. This can result from different positions of the magnification device in the sagittal plane. These small changes in positioning the device should be avoided to achieve accurate deformity correction and bone lengthening. This should be considered for all length and size measurements on radiographs.

## Introduction

Long-leg weight-bearing radiographs (LLR) provide a precise view of the whole lower limb including the centre of the femoral head, the knee and the ankle. In clinical practice as well as in clinical studies, LLR are routinely obtained pre- and postoperatively for bone deformity corrections, knee osteotomies, knee replacements, and to control limb lengthening or a segmental bone transport [1–7]. Using LLR, it is possible to measure mechanical and anatomical limb alignment and determine limb length [8, 8].

**Fig. 1:**
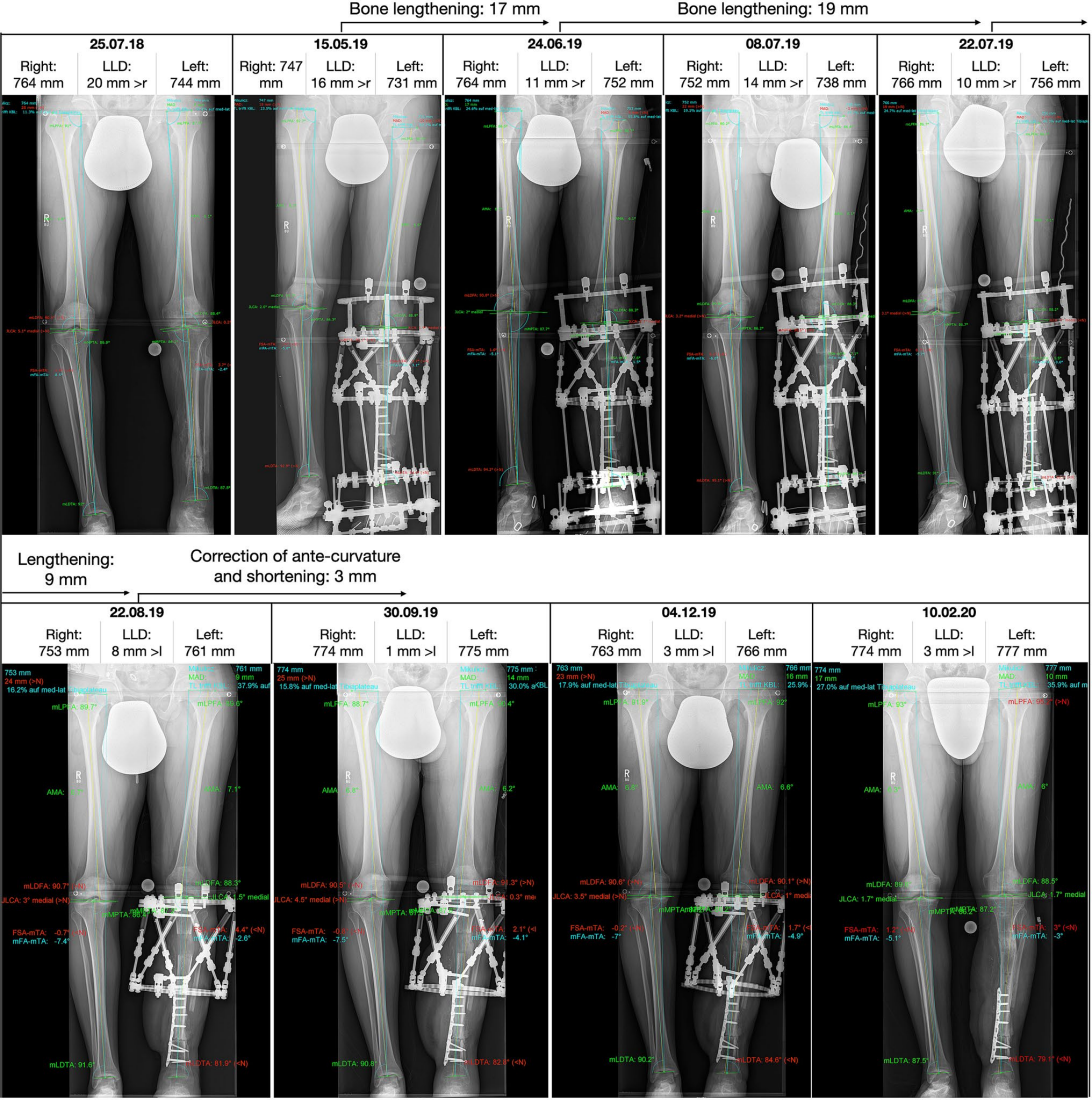
Example of the variability of limb length within one patient during bone lengthening. The length of the untreated right leg varies between 747 and 774 mm. Bone lengthening protocols had to be re-calculated due to variability of limb length of both legs on long-leg radiographs leading to prolonged treatment time (approx. 3 months) (*mFA-mTA* mechanical tibiofemoral angle, *MPTA* medial proximal tibia angle, *mLDFA* mechanical lateral distal femur angle, *mLDTA* mechanical lateral distal tibia angle, *mLPFA* mechanical lateral proximal femur angle, *JLCA* joint line convergence angle, *AMA* anatomical and mechanical femoral angle)

To treat patients with bone lengthening procedures or bone segment transport, the Taylor Spatial Frame (TSF, Smith & Nephew, Memphis, USA) is one of a variety of treatment options to restore bone integrity [10]. In this setting, the TSF is an accurate treatment device to promote healing of complex nonunions with multiplanar deformities and significant mechanical axis deviation [11]. In these patients, the contralateral uninjured leg is used as a reference to determine limb length discrepancies on LLR. However, during bone transport procedures, surgeons often deal with the problem that the measured limb length of the contralateral side varies between each LLR of the same patient. A clinical example is presented in Fig. 1. Due to these measured limb length inconsistencies and despite precise planning on the pre-operative LLR as well as an accurate bone transport, remaining length discrepancies and remaining deformities can occur after the initially planned deformity or length correction [11–14]. Iterative adjustment of the bone transport and the deformity correction based on the control radiographs are often necessary until the desired mechanical axis alignment is achieved [11, 15]. These adjustments are time-consuming, result in higher radiation exposure for the patient, increase treatment time of bone lengthening procedures with increasing risk of pin infections and finally lead to patient’s and doctor’s dissatisfaction. So far, studies and knowledge are limited regarding the variability of the measured limb length on LLR.

Therefore, the primary aim of the study was to examine the intra-individual variability of the measured limb length on clinically indicated LLR. Only patients undergoing single-side TSF treatment were included as they usually receive several LLR during bone deformity correction and intra-individual variability of the healthy contra-lateral side could be analysed without the need for additional radiographs. We hypothesized that the differences of measured limb length between LLR in one patient are higher than the differences of the measured limb length between observations and observers.

The secondary aim was to examine the influence of the position, size and calibration technique of a magnification device on measured limb length on LLR using a radiographic test setup.

## Material and methods

### TSF-patients and radiographs taken during deformity correction

Between 2016 and 2020, 44 patients underwent deformity correction using a TSF in our clinic. The study retrospectively measured the length of the healthy, untreated limb on LLR of these patients. All radiographs were conducted for clinical reasons and not for the purpose of this study or other related research. The local ethical committee approved the study (894/2018BO2).

Patients who additionally underwent surgery on the “healthy” contralateral leg (*n* = 1) or with less than four LLR (*n* = 5) during TSF-treatment were excluded. Radiographs without a magnification device were excluded (*n* = 12). The final data set comprised 38 patients [46.2 ± 13.4 years (21.3–80.8)]. TSF-treatment was performed due to multiplanar posttraumatic bone deformity of the tibia with and without bone infection by one consultant [16]. Bone lengthening was performed in 33 out of these 38 patients. Patient demographics are reported in Table 1.

**Table 1:** Patient demographics

Demographics	
Gender	Female: 2 (5.3%) Male: 36 (94.7%)
Age (years)	46.2 ± 13.4 (21.3–80.8)
Body height (cm)	176.1 ± 7.7 (160–194)
Body weight (kg)	88.4 ± 19.1 (52–130)
Body mass index (kg/m^2^)	28.5 ± 5.5 (17.9–41.5)
TSF treatment	Deformity correction: 38 (100%) Bone infection: 21 (55.3%) Limb lengthening 33 (86.8%) Segment transport: 8 (21.1%)

LLR were obtained in accordance with Paley [8]. The distance from the radiographic tube to the film was 305 cm. The X-ray beams were centred on the level of the knee joints. A magnification device (25 mm steel ball) was used to calibrate the radiographs.

### Radiographic test setup

In the test setup, LLR were obtained in the same manner as usually used for routine assessment in the daily practice as described above.

**Fig. 2:**
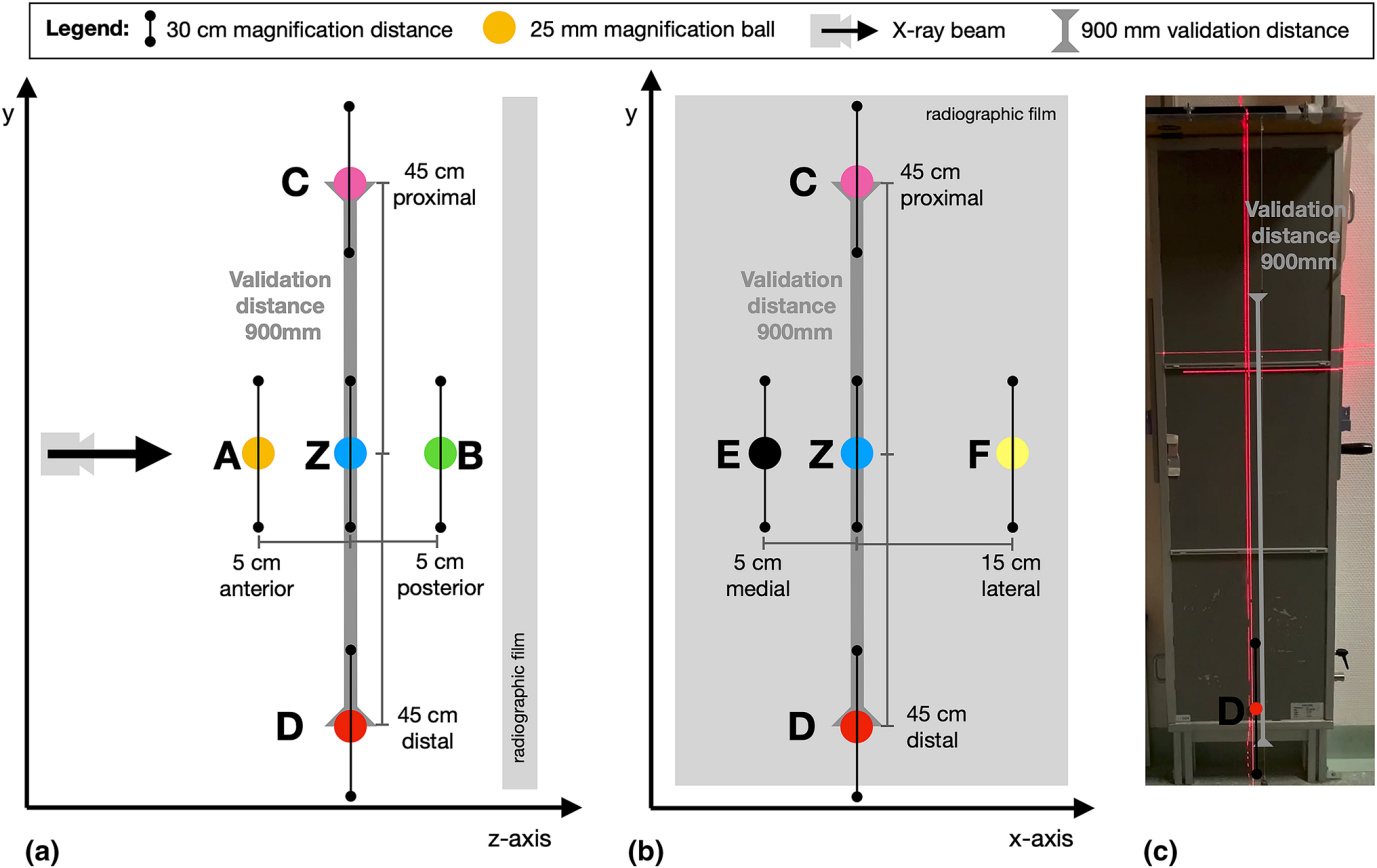
Overview of the radiographic test setup. **a** Sagittal view: the X-ray source is 295 cm away from the validation distance. The *z*-position of the magnification cord (including steel ball and magnification distance) is in the middle of the validation distance. Position A is 5 cm anterior, position B 5 cm posterior of the *z*-position. On the same sagittal level (*z*-axis) as the validation distance and the *z*-position, position C is 45 cm proximal and position D is 45 cm distal from the *z*-position. **b** Frontal view: the position E is 5 cm medial and position F is 15 cm lateral from the *z*-position. Positions E, Z and F are on the same sagittal level as the validation distance. **c** Frontal view of the radiographic film with the magnification cord and the validation cord

The test setup (Fig. 2) consisted of a metal plate with holes in defined distances and positions to each other. In these holes, a validation cord and a magnification cord were hanged in to provide that the cords were freely suspended in defined positions. The validation cord had two iron markers (diameter 3 mm) with a distance of 900 mm to each other (validation distance). The magnification cord consisted of a magnification distance and a 25-mm-steel ball. The steel ball was placed in the middle of the magnification distance. The magnification distance had a length of 300 mm which was marked at each end with an iron marker.

The validation cord was placed 295 cm away from the X-ray source, 10 cm in front of the X-ray film. The central X-ray beam was centered to the middle of the validation distance. In relation to the validation cord, the magnification cord was placed in seven different positions (Fig. 2): The central position (*z*-position) of the magnification cord was on the same sagittal level (295 cm away from the X-ray source) as the validation distance. The magnification cord was placed in a manner that the magnification ball and subsequently the middle of the magnification distance were in the middle of the validation distance. Along the X-ray beam, position A was 5 cm anterior, position B was 5 cm posterior in relation to the *z*-position. On the same sagittal level than the validation cord, position C of the magnification cord was defined as 45 cm proximal and position D is 45 cm distal to the *z*-position. Position E was defined as 5 cm medial to the *z*-position, parallel to the film. Position F was defined as 15 cm lateral to the *z*-position.

For each position of the magnification cord, an LLR was taken from the test object including the validation cord and the magnification cord.

### Measurements

The radiographs to determine limb length of the TSF patients as well as the radiographs taken for the radiographic tests were measured using mediCAD® (Hectec, Landshut, Germany). All radiographs were measured twice by two observers (orthopaedic residents with at least 4 years of experience in musculoskeletal imaging) independently.

Several LLR radiographs of TSF patients could not be automatically calibrated by mediCAD software because of interference with the TSF hardware. Therefore, all radiographs of TSF patients were calibrated manually by using the mediCAD function “calibration with a circle defined by three points”. The limb length was defined as the distance between the center of the femoral head and the centre of the ankle joint line (middle between the medial and lateral border of the talus dome).

All radiographs of the radiographic test setup were calibrated both automatically and manually using the 25 mm magnification ball. Additionally, manual calibration was performed using a 30 cm magnification distance.

### Statistics

To answer the primary aim of our study, the following statistical analyses were performed: the absolute values of the length of the untreated limb were used. The mean, standard deviation (SD) and the range (maximum–minimum) of the measured limb lengths were calculated for each patient independently. The range and SD of the measured limb length on LLR of each individual patient were used to describe the variability of measured limb length within a patient. The average variability of these ranges and SD were calculated for the total cohort [mean ± SD (minimum–maximum)].

To answer the secondary aim of our study, descriptive statistics were calculated to describe the variability of measured distances dependent on the position of the magnification device as well as dependent on the calibration method (automatic calibration with the 25 mm magnification ball, manual calibration with the 25 mm magnification ball, manual calibration with the 30 cm magnification distance). To find out differences dependent on the position of the magnification device, averages [mean ± SD (minimum–maximum)] of all measurements for each position were calculated. Differences in lengths between the three respective positions (anterior vs. central vs. posterior, medial vs. central vs. lateral, proximal vs. center vs. distal) were calculated. Differences of the measured object length were intrepreted taking inter-observer and intra-observer measurement differences into account.

The intra- and interrater reliability were determined by calculating the intra-class correlation coefficient (ICC) for measurements of the primary and secondary study aim. The ICC gives a value between 0 and 1 and describes the correlation among pairs of data. ICC and 95% confidence intervals were based on a two-way mixed-effects model [ICC (3,1)]. The ICC values were interpreted as suggested by Shrout and Fleiss [17]. An ICC above 0.75 indicates excellent reliability.

The study data were analysed with JMP® (SAS Institute Inc., 14.2, Cary, NC, USA) and STATA® (Stata Corporation, 15.0, College Station, TX, USA).

## Results

The data set comprised 38 patients and 278 LLR in total. In average, 7.3 ± 2.6 (4–13) LLR were taken from one patient.

The measured length of the healthy, uninjured limb showed high intra-individual variability (Table 2). The intra-individually measured limb length varied in all patients by at least 1 cm between LLR. 25 (65.8%), 26 (68.4%), 26 (68.4%) and 29 (76.3%) patients had a variation of ≥ 2 cm between LLR (first and second observation of observer 1 and 2). The measured limb length between LLR ranged between 11–43 mm (observer 1, first observation), 12–50 mm (observer 1, second observation), 10–42 mm (observer 2, first observation) and 10–47 mm (observer 2, second observation) within one patient. Relatively to the individuals’ mean limb length, the variability of limb length between LLR ranged between 1.2% and 6.5%.

**Table 2:** The mean ± standard deviation (range) of the limb lengths measured throughout all LLR (*), the mean ± standard deviation (range) of the ranges of the measured limb length within a single patient (^#^), the mean ± standard deviation (range) of the standard deviations of the measured limb length within a single patient (°)

	Mean limb length of the cohort (*)	Ranges of measured limb length within each patient (^#^)	Deviations (SD) of measured limb length within each patient (°)
Observer 1			
Observation 1	833.6 ± 48.4 (748–926) mm	23.74 ± 8.09 (11–43) mm	8.41 ± 2.79 (3.97–16.38) mm
Observation 2	833.9 ± 48.7 (747–924) mm	24.24 ± 8.45 (12–50) mm	8.57 ± 2.76 (3.92–16.34) mm
Observer 2			
Observation 1	831.6 ± 48.1 (745–924) mm	25.47 ± 8.60 (10–42) mm	9.07 ± 2.90 (4.20–17.17) mm
Observation 2	832.7 ± 48.0 (747–923) mm	25.08 ± 8.96 (10–47) mm	9.00 ± 2.81 (4.10–17.90) mm

The ICC showed excellent intra- and interobserver reliability with small measurement differences between observers (4.17 ± 3.16; 3.75 ± 2.97 mm) and between observations (2.78 ± 2.01; 4.07 ± 3.28 mm) (Table 3).

**Table 3:** Intra- and interobserver reliability [ICC (95% CI)] and intra- and interobserver difference (mean ± standard deviation (minimum–maximum)) of limb length measurements in TSF patients

	Intraobserver reliability	Intraobserver differences—absolute values	Interobserver reliability	Interobserver differences—absolute values
Limb length measurement in TSF patients	Observer 1: 0.998 (0.997–0.998)	Observer 1: 2.78 ± 2.01 (0–10) mm	First observation: 0.995 (0.994–0.996)	First observation: 4.17 ± 3.16 (0–16) mm
	Observer 2: 0.995 (0.993–0.996)	Observer 2: 4.07 ± 3.28 (0–16) mm	Second observation: 0.996 (0.994–0.996)	Second observation: 3.75 ± 2.97 (0–13) mm

**Fig. 3:**
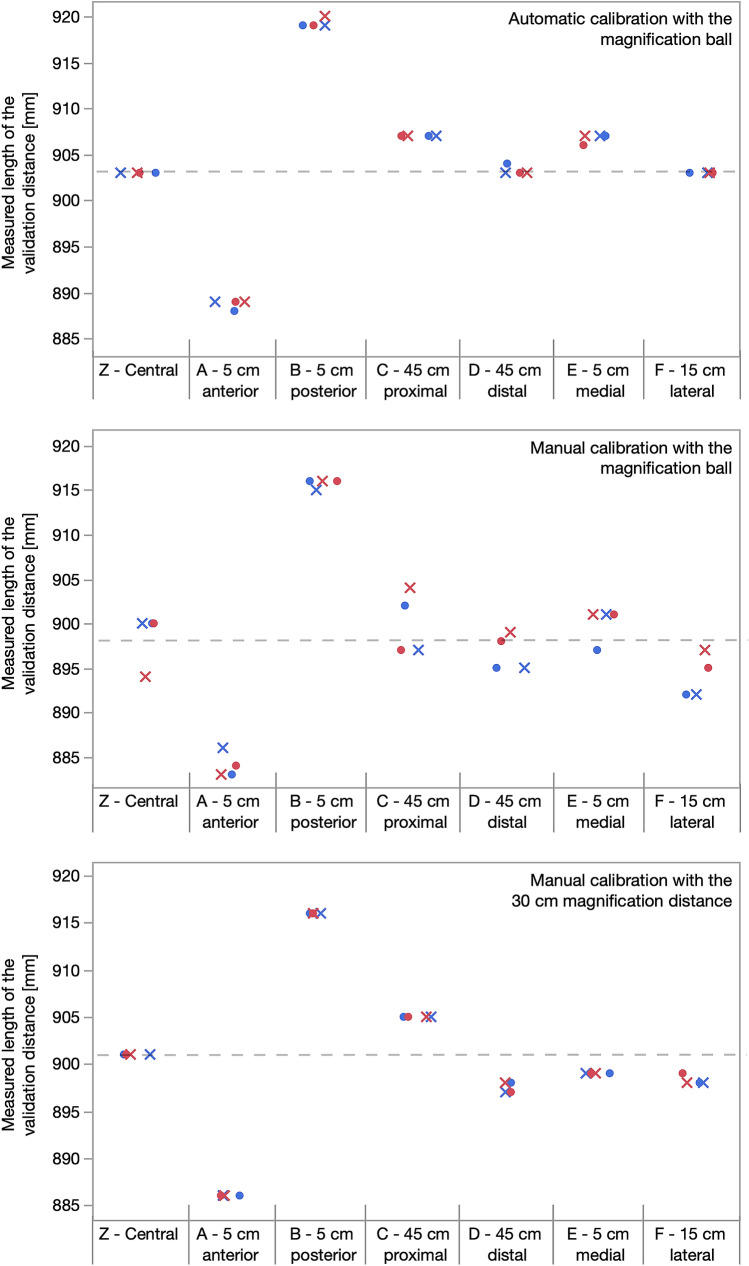
The measured length of the validation distance differed regarding the position of the magnification device. Automatic and manual calibration with the 25-mm magnification ball and manual calibration with the 30 cm magnification distance were presented seperately. Manual calibration with the 25-mm magnification ball showed the largest variation between measurements (dot: observer 1, x: observer 2, blue colour indicates first and red colour indicates second observation)

The results of the secondary aim showed length variability depending on the position of the magnification device. A change of position in the sagittal plane had the largest effect on the measured length (Fig. 3, Table 4): Positioning the magnification device 5 cm (position A) anterior to the 90 cm validation distance led to smaller measurements (14.6 mm) compared to the central z-position. Positioning the magnification device 5 cm posterior to the object (position B) led to larger measurements (16.2 mm) compared to the z-position. The measured length of the validation distance with a 45 cm proximal (position C 3.2 mm), 45 cm distal (position D 1.7 mm), 5 cm medial (position E 1.1 mm) or 15 cm lateral (position F 2.4 mm) position of the magnification device differed not relevantly compared to the measured length of the validation distance with the device in the z-position.

**Table 4:** Measured object length at different positions of the magnification device

Positions	Mean ± SD (min.–max.)	Mean differences between positions
(A) 5 cm anterior	886.3 ± 2.2 (883–889)	Center vs. anterior: − 14.6 mm
(Z) Center	900.8 ± 2.5 (894–903)	Center vs. posterior: 16.2 mm
(B) 5 cm posterior	917.0 ± 1.7 (915–920)	Anterior vs. posterior: 30.8 mm
(C) 45 cm proximal	904.0 ± 3.6 (897–907)	Center vs. proximal: 3.2 mm
(Z) Center	900.8 ± 2.5 (894–903)	Center vs. distal: − 1.7 mm
(D) 45 cm distal	899.2 ± 3.2 (895–904)	Proximal vs. distal: − 4.8 mm
(E) 5 cm medial	901.9 ± 3.8 (897–907)	Center vs. medial: 1.1 mm
(Z) Center	900.8 ± 2.5 (894–903)	Center vs. lateral: − 2.4 mm
(F) 15 cm lateral	898.4 ± 4.1 (892–903)	Medial vs. lateral: − 3.5 mm

All measurements regardless of observer, observation or calibration method were combined and compared to the other postions

Smaller inter- and intraobserver differences were found when measurements were performed with automatic calibration (≤ 1 mm) compared to manual calibration (≤ 7 mm) using a 25-mm magnification ball. Inter- and intraobserver differences with manual calibration using the 30 cm magnification distance were ≤ 1 mm. ICC showed excellent intra- and interobserver reliability (Table 5).

**Table 5:** Intra- and interobserver reliability (ICC (95%CI)) and intra- and interobserver difference [mean ± standard deviation (minimum–maximum)] of limb length measurements in the test setup with automatic and manual calibration with the 25 mm magnification ball and manual calibration with the 30 cm magnification distance

	Intraobserver reliability	Intraobserver differences—absolute values	Interobserver reliability	Interobserver differences—absolute values
Test setup				
Automatic calibration with the magnification ball	Observer 1: 0.999 (0.995–1.000)	Observer 1: 0.1 ± 0.4 (0–1) mm	First observation: 0.998 (0.988–1.000)	First observation: 0.3 ± 0.5 (0–1) mm
	Observer 2: 0.997 (0.983–0.999)	Observer 2: 0.4 ± 0.5 (0–1) mm	Second observation: 0.999 (0.991–1.000)	Second observation: 0.3 ± 0.5 (0–1) mm
Manual calibration with the magnification ball	Observer 1: 0.884 (0.472–0.980)	Observer 1: 3.7 ± 2.6 (0–7) mm	First observation: 0.954 (0.759–0.992)	First observation: 1.9 ± 2.1 (0–5) mm
	Observer 2: 0.952 (0.782–0.992)	Observer 2: 2.3 ± 2.0 (0–5) mm	Second observation: 0.921 (0.616–0.986)	Second observation: 2.4 ± 2.9 (0–7) mm
Manual calibration with the 30 cm magnification distance	Observer 1 0.999 (0.995–1.000)	Observer 1 0.1 ± 0.4 (0–1) mm	First observation: 0.999 (0.995–1.000);	First observation: 0.1 ± 0.4 (0–1) mm
	Observer 2: 0.998 (0.988–1.000)	Observer 2: 0.3 ± 0.5 (0–1) mm	Second observation: 0.998 (0.988–1.000);	Second observation: 0.3 ± 0.5 (0–1) mm

## Discussion

We found high variability of limb length between taken radiographs within one patient. Discrepancies between radiographs ranged from 10 mm up to 50 mm within one patient. All patients had limb length differences of more than 1 cm and up to 76% of patients even had a measured limb length difference of more than 2 cm. As hypothesized, these measured limb length differences within one patient are higher than measurement differences found between observers and observations. The secondary aim of our study revealed that these observed differences can be explained by different positioning of the magnification device. Large influence was observed when changing the position in the sagittal plane. Placing the magnification device just 5 cm anterior or posterior to the reference, central position (Z), the measured length changed signficantly in average − 15 and + 16 mm. These small changes in positioning the maginfication device can critically influence the deformity planning and correction, especially when bone lengthening is required, as demonstrated in Fig. 1.

Limb length discrepancy between both legs was found to affect function of the lower extremity as well as quality of life [18–21]. Moraal et al. [22] reported that long-term quality-of-life scores following limb lengthening in children were similar to those of controls, especially when the remaining limb-length discrepancy was smaller than 2 cm. Associations between radiographically measured limb length discrepancy and low back pain [23], knee osteoarthritis knee [24], inferior functional outcomes and patient satisfaction following total hip or knee replacement were described [18, 19, 25]. A discrepancy of > 1 cm leads to a higher risk of knee osteoarthritis of both legs and an altered gait [20, 24].

In our study, we did not measure limb length discrepancies between left and right leg, because one leg was always treated for bone deformity including limb length corrections with a TSF. In contrast, we analyzed leg length differences between radiographs within each patient. During bone lengthening, transport and deformity correction, the contralateral, untreated leg is usually used as the reference to perform deformity corrections and to adjust the limb length [8]. However, as found in our study the measured length of the contralateral healthy leg ranged in average between 11 and 50 mm in each individual. This difference is significantly higher than the observed measurement inaccuracy between observations (intra-observer variability) and different observers (inter-observer variability) in our study. To the best of our knowledge, there is no previous study reporting this high variability of measured limb length between LLR of the same patient. The measured length differences between each LLR can lead to inaccurate bone transports. A residual deformity occurs in approximately one third of the patients after the initial correction [11–14]. The advantage of TSF treatment is that new protocols can be calculated to adjust for these measurement errors. With the use of other internal implants such as intra-medullary bone transport nails, these adjustments cannot be made. However, even in TSF treatment these measured leg length differences result in a longer duration of bone transport leading to a dissatisfaction of patients and surgeons as well as higher radiation exposure as more LLR haven to be taken to evaluate progress of bone correction treatment.

**Fig. 4:**
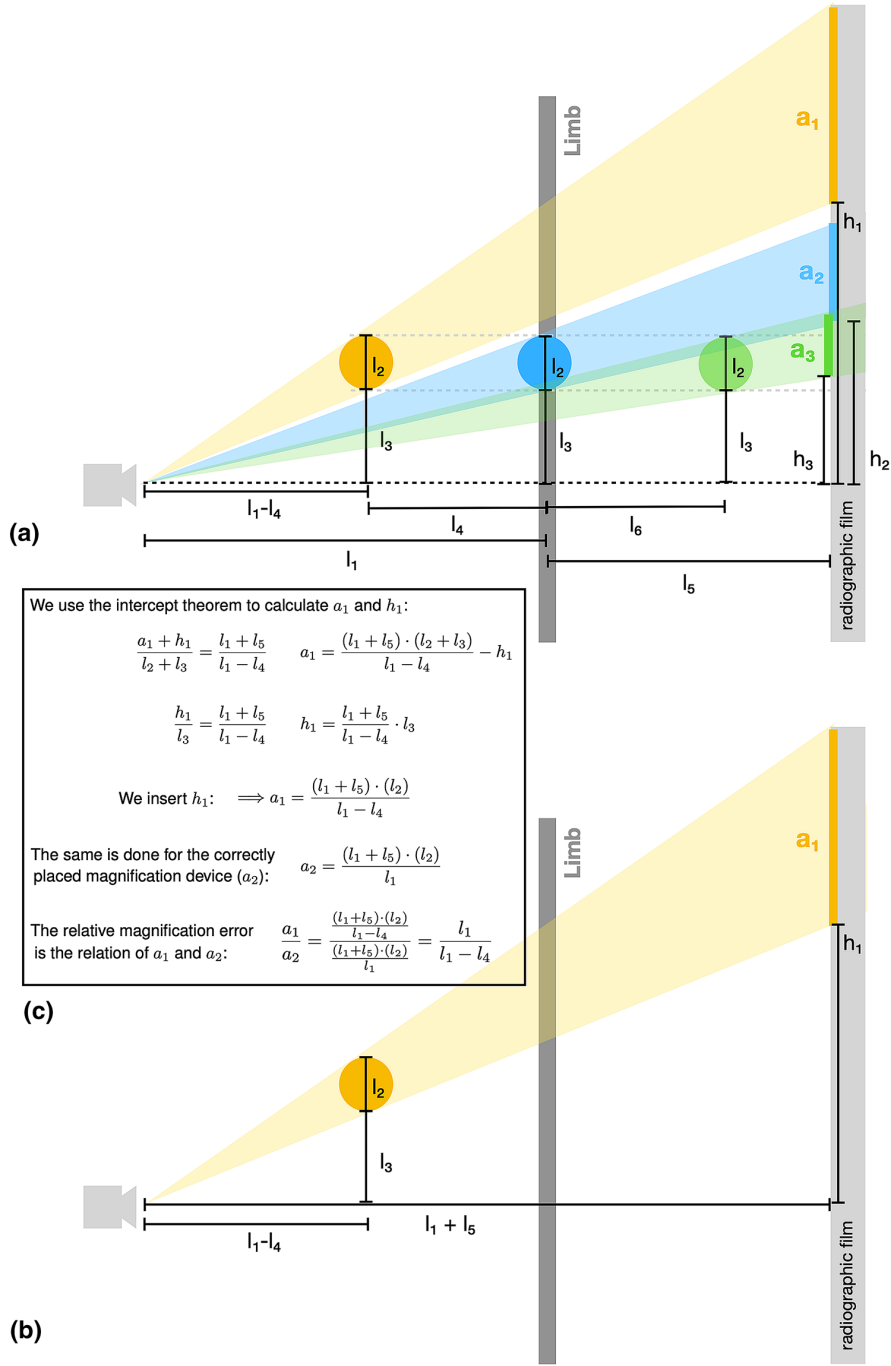
**a** Overview of all distances in the test setup. **b** Focused on the relevant lines for calculating the depicted magnification device on the radiograph (*a*_1_). **c** Mathematical explanation, why correct magnification device positioning is important to avoid magnification errors. The final formula consists of the distance from the X-ray tube to the correct position/same level of the limb (*l*_1_) and the distance from X-ray tube to position of the magnification device (*l*_1_–*l*_4_). The size of the magnification device (*l*_2_), the level of the device in the frontal plane (*h*_1_, *l*_3_) and the distance from leg to radiographic film (*l*_5_) can be neglected and do not influence magnification errors mathematically

The high variability of measured limb length between LLR could be explained by altered positions of the magnification device. We found in the radiographic test setup that the position of the magnification device in the sagittal plane has a significant impact on the measured leg length. Different positions of the device in the frontal plane (proximal–distal or lateral-medial) had a smaller impact on the limb length than those in the sagittal plane. The impact of these different positions in the frontal plane was comparable to the inter- and intra-observer measurement differences if manual calibration of the 25 mm steel ball was performed. Measured differences due to altered sagittal position of the magnification device can be mathematically explained as shown in Fig. 4 using the intercept theorem. The final formula consists of the distance from the X-ray tube to the correct position/same level of the limb (*l*_1_) and the distance from X-ray tube to position of the magnification device used as a reference (*l*_1_–*l*_4_). The size of the magnification device (*l*_2_), the level of the device in the frontal plane (*h*_1_, *l*_3_) and the distance from the leg to the detector film (*l*_5_) can be neglected and do not influence magnification errors in a mathematic approach. However, in practice the size of the magnification device (*l*_2_) leads to higher measurement inaccuracy and *l*_5_ can only be neglected if both legs are in the exact same position in the sagittal plane. Applying this formula to the magnification device position A, the device is depicted too large on the radiographic film by a factor of 1.017 (295 cm divided by 200 cm). Resultingly, the leg length equals 885 mm (900 mm divided by the magnification factor 1.017).

LLR are commonly used and are considered to be a reliable method for deformity analysis in the frontal plane as well as limb length measurements [8, 9, 20]. The accuracy of this method is defined by the variation to the actual limb length. Sabharwal et al. [9] reported about a magnification error of approximately 5%, which is dependent on the length and girth of the limb, the distance of the X-ray source to the cassette and divergence of the X-ray beam. By placing a magnification marker next to the patient, this magnification error can be reduced [9]. Sabharwal et al. [26] analysed intraobserver and interobserver reliability among five blinded observers with varying degrees of experience assessing leg length discrepancies on standing radiographs of 70 patients. The intraobserver (ICC 0.939–0.996) and interobserver reliability (ICC 0.968) for all five observers were high. The mean absolute difference was 1.5–4.6 mm for intraobserver reliability, and 3 mm for interobserver reliability. Schröter et al. [27] analysed the reliability of digital planning of 81 high tibial osteotomies using 81 LLR. High interobserver reliability (ICC 0.981; 0.974) was found to measure limb length with the software PreOPlan and MediCAD. We found similar reliability compared to Sabharwal et al. [26] and Schröter et al. [27].

Besides the position of the magnification device, the calibration method influences the measured leg length, too. Automatic calibration using the dedicated software showed nearly identical measurements for different observations and observers. Unfortunately, due to the TSF hardware the magnification device (25 mm steel ball) cannot be automatically calibrated in approximately one-third of the cases leading to higher measurement inaccuracy when manually calibrated has to be performed. This has to be taken into account when interpreting the results of the first aim of our study. Higher measurement reliability, similar to automatic calibration with the steel ball, can be achieved using a 30 cm, freely suspended magnification distance, even though it is also calibrated manually. However, these measurement inaccuracies are small compared to the differences found between measured limb length on different radiographs within one TSF-patient.

Based on our findings, we recommend placing the magnification device in the correct sagittal and in a reproducible position. The device should be placed medial of the femoral condyles of the untreated limb. In patients with ring fixators, the hardware can be used to reproduce the positions of the magnification device. If automatic calibration is not possible due to hardware interference with the steel ball, a 30 cm, freely hanging magnification distance can be used to caliber radiographs and to decrease measurement errors. In addition, attention has to be paid to position the limb in neutral rotation with the patella pointing forward. External rotation causes less apparent valgus and leads to more varus. Internal rotation pretends more valgus and leads to less varus [28–33]. In patients with a circular fixator, limbs are positioned more in external rotation than without an external fixator [33]. To achieve higher reliability and to reduce malpositioning of the limb on LLR, a rod can be placed at the reference ring of the external fixator to better control limb positioning [34]. Moreover, the use of the light source and the laser from the X-ray can enhance the quality of radiographs [13, 35].

The main limitation of the study is that we evaluated measured leg length of one limb, but could not determine leg length discrepancies between ipsilateral and contralateral side since all patients had unilaterally an altered leg length due to TSF treatment. Errors in determining the size of the magnification object may influence the measure length of both limbs with the same extent and thus may result only in absolute errors of limb length but not in leg length discrepancies. Further limitations are the retrospective nature of the study design and that no power analysis was calculated prior to the study. Last but not least, LLR is a two-dimensional image modality depicting a three-dimensional deformity. Three-dimensional analysis using imaging modalities such as weight-bearing CT scans [36, 37] or the biplanar linear radiograph system (EOS®) [1, 38–40] are alternatives to analyze the deformities three dimensionally under weight-bearing. However, these techniques are currently not routinely available in most clinics. EOS captures an anteroposterior and a lateral radiograph simultaneously of the whole lower limb during weight-bearing [38]. EOS® allows three-dimensional reconstruction and accurate measurements as well as having lower radiation dose and lower error rates due to malrotation of the lower limb [1, 38, 39, 41, 42]. Further studies are needed to improve accuracy of radiological measurement using LLR and to draw conclusions about the best imaging technique for surgical planning of deformity correction.

## Conclusions

High variability of limb length ranging from 1 to 5 cm between different LLR within one patient was observed. This variability is higher than absolute difference of inter- and intra- observer reliability. The high variability can be explained by different positions of the magnification device in the sagittal plane. Placing the device 5 cm anterior or posterior of the limb, the measured length changed signficantly on LLR. Surgeons, radiologists and radiology assistants have to place the magnification device in the correct and in a reproducible position. Moreover, measurement inaccuracy can be reduced if automatic calibration with the magnification ball using the planning software or manual calibration with a 30 cm magnification distance is used. Our results are applicable not only to patients treated with TSF. Our findings should be taken into account for all size and length measurements on radiographs.
